# Leakage Identification of Underground Structures Using Classification Deep Neural Networks and Transfer Learning

**DOI:** 10.3390/s24175569

**Published:** 2024-08-28

**Authors:** Wenyang Wang, Qingwei Chen, Yongjiang Shen, Zhengliang Xiang

**Affiliations:** 1 Shandong Zhiyuan Electric Power Design Consulting Co., Ltd., Jinan 250021, China; sdhhwwy@126.com (W.W.); chenqingwei@tju.edu.cn (Q.C.); 2 Economic & Technology Research Institute of State Grid Shandong Electric Power Company, Jinan 250021, China; 3 Hunan Province Key Laboratory for Disaster Prevention and Mitigation of Rail Transit Engineering Structure, Central South University, Changsha 410075, China; 209059@csu.edu.cn; 4 School of Civil Engineering, Central South University, Changsha 410075, China

**Keywords:** underground structures, water leakage defect, computer vision, transfer learning, deep learning

## Abstract

Water leakage defects often occur in underground structures, leading to accelerated structural aging and threatening structural safety. Leakage identification can detect early diseases of underground structures and provide important guidance for reinforcement and maintenance. Deep learning-based computer vision methods have been rapidly developed and widely used in many fields. However, establishing a deep learning model for underground structure leakage identification usually requires a lot of training data on leakage defects, which is very expensive. To overcome the data shortage, a deep neural network method for leakage identification is developed based on transfer learning in this paper. For comparison, four famous classification models, including VGG16, AlexNet, SqueezeNet, and ResNet18, are constructed. To train the classification models, a transfer learning strategy is developed, and a dataset of underground structure leakage is created. Finally, the classification performance on the leakage dataset of different deep learning models is comparatively studied under different sizes of training data. The results showed that the VGG16, AlexNet, and SqueezeNet models with transfer learning can overall provide higher and more stable classification performance on the leakage dataset than those without transfer learning. The ResNet18 model with transfer learning can overall provide a similar value of classification performance on the leakage dataset than that without transfer learning, but its classification performance is more stable than that without transfer learning. In addition, the SqueezeNet model obtains an overall higher and more stable performance than the comparative models on the leakage dataset for all classification metrics.

## 1. Introduction

With the rapid development of urban areas all over the world, many engineering structures are constructed underground, such as metro tunnels [1], underground garages [2], underground substations [3], etc. Underground structures are usually affected by many adverse internal and external effects, including corrosion, groundwater, and operation loads. These effects may lead to the performance deterioration of the underground structures. Water leakage is a very common disease of underground structures, which can accelerate structural aging and cause serious structural safety accidents. Therefore, it is very important to conduct water leakage detection for underground structures during the operation process.

Conventional manual detection methods require a lot of human and material resources, and efficiency is low. In recent years, structural health monitoring has been studied and applied widely [4,5,6,7,8,9]. Using advanced sensors and data mining algorithms, structural health monitoring systems can provide timely condition information on structures. Compared with the conventional manual detection method, the detection results of structural health monitoring are more objective, labor saving, and cost-effective [10]. There are many kinds of sensors used in structural health monitoring, which can obtain different condition information on the monitoring objectives. Generally, these sensors can be divided into contact and non-contact sensors. Contact sensors need to be in contact with the monitoring object to collect information on its changes, including the physical quantities such as pressure, stress, strain, and temperature. When using a non-contact sensor, physical contact with the monitoring object is not required, and the data on the monitoring object can be measured by measuring changes in the electric field, magnetic field, light, sound, etc. In recent years, as a kind of non-contact sensor, the vision sensor has been widely used in civil engineering structure health monitoring [11,12,13,14,15,16]. In the field of leakage identification of underground structures, many computer vision techniques also have been proposed for the classification and location of leakage defects [17,18,19,20]. These computer vision techniques generally can be divided into four categories, including image classification [21], object detection [22], semantic segmentation [23], and instance segmentation [24]. The image classification method is used to identify whether there is a leakage defect in the images. Object detection methods can be used to detect the leakage defect in the images and locate the regions. The semantic segmentation method can classify the pixels of different defects in the images. At last, the instance segmentation method can classify the pixels of different items of each defect in the images. Compared with the other three methods, the image classification method generally needs lower computational cost and is ideal for fast leakage defect detection.

With the development of artificial intelligence, many deep learning-based computer vision methods have been proposed. These methods have been validated to be more powerful than conventional computer vision methods. The VGG model [25], AlexNet model [26], SqueezeNet model [27], and ResNet model [28] are four very famous deep learning-based classification models that can provide high-accuracy classification results for many datasets. However, most deep learning models need a lot of training data to ensure classification performance. If there is insufficient training data to train the deep neural network, the accuracy of leakage identification will be low. To deal with the insufficient training data problems, transfer learning [29,30,31], few-shot meta-learning [32,33], semi-supervised learning [34,35], active learning [36,37,38], and other advanced methods have been proposed recently. In these methods, the latter several methods usually use carefully designed network structures or training data selection strategies to improve the classification performance of the deep neural network. But, the training data are restricted to the limited objective dataset. However, the transfer learning methods take advantage of the related datasets, which may be large enough to pretrain the deep neural network of classification models. The pretrained deep neural network has a strong ability to extract the features of the images. Then, much smaller training data in the objective dataset is needed to retrain the deep neural network. Because the transfer learning method can use the information from both the training data of the objective dataset and the related datasets, and the size of the related datasets is not limited, the transfer learning method greatly saves the training data in objective dataset. Recently, transfer learning has attracted some attention in the field of defect detection of engineering structures [39,40,41]. However, most of the references only consider one classification model. How transfer learning affects the performance of different deep learning-based classification models for leakage identification of underground structures is unclear.

In this paper, a leakage identification method for underground structures is developed based on the classification of deep neural networks and transfer learning. The main contributions of this paper are as follows. (1) The performance of the classification models incorporating transfer learning is validated in the leakage identification of underground structures and (2) the influence of transfer learning on different classification models (including the VGG16 model, AlexNet model, SqueezeNet model, and ResNet model) and the influence of training data size on their classification performance are comparatively studied.

## 2. Methodology

### 2.1. Deep Neural Network for Leakage Identification

In this paper, four deep neural networks for leakage identification were studied, which were the VGG16 model, AlexNet model, SqueezeNet model, and ResNet model. All pretrained models were downloaded from the Torchvision library. The input size of the model was 224 × 224 × 3. To classify the leakage dataset, the output size of the models was changed to 1 × 1 × 2.

**Figure 1:**
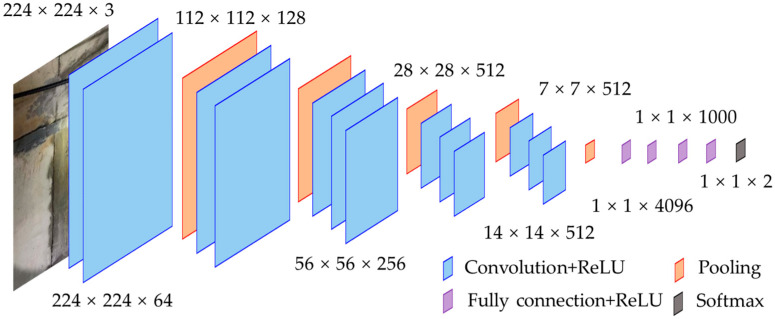
Modified VGG16 model.

The VGG (Visual Geometry Group) model is a deep convolutional neural network architecture proposed by the Visual Geometry Group in 2014. The architecture of the modified VGG16 model is shown in Figure 1. The original VGG16 model had 16 weight layers, including 13 convolutional layers and 3 fully connected layers. To classify the leakage defect, a fully connected layer with two outputs was added to the pretrained model. VGG16 has a simple network structure and is easy to implement, making it one of the classic models in the field of deep learning-based computer vision. Due to the deep convolutional structure, the number of parameters in the VGG model is very large.

**Figure 2:**
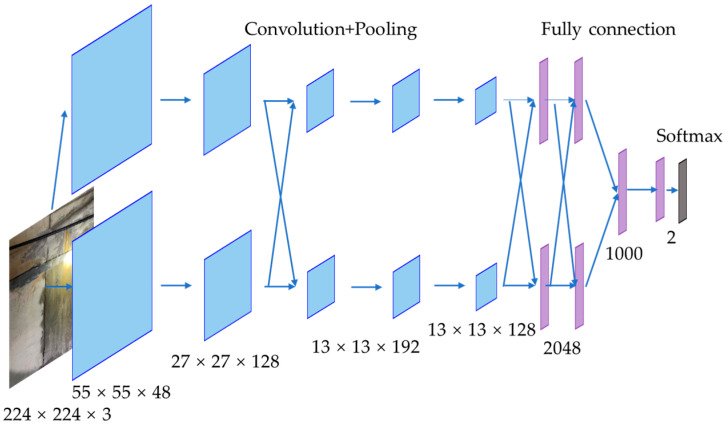
Modified AlexNet model.

AlexNet is a classic convolutional neural network, and it was proposed in the 2012 ImageNet image classification competition. The architecture of the modified AlexNet model is shown in Figure 2. As shown in this figure, the modified AlexNet model had five convolutional layers and four fully connected layers, where the last fully connected layer was added to change the output classes. AlexNet was an early implementation of a deep convolutional neural network. By increasing the network depth, AlexNet was able to better learn the features of the dataset, thereby improving the accuracy of image classification.

**Figure 3:**
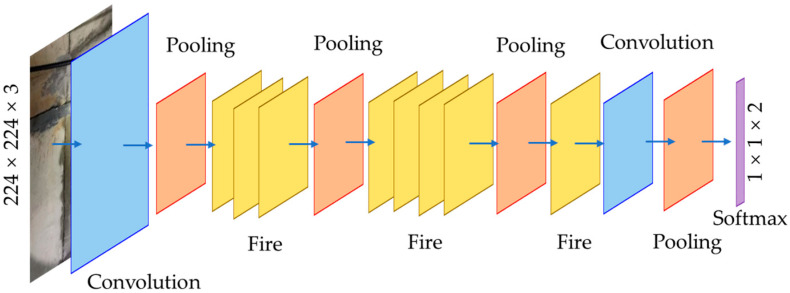
Modified SqueezeNet model.

SqueezeNet is a lightweight deep learning model that can achieve high prediction accuracy with fewer model parameters. Figure 3 shows the architecture of the modified SqueezeNet model. The modified SqueezeNet model had two convolutional layers, four pooling layers, and eight fire modules.

**Figure 4:**
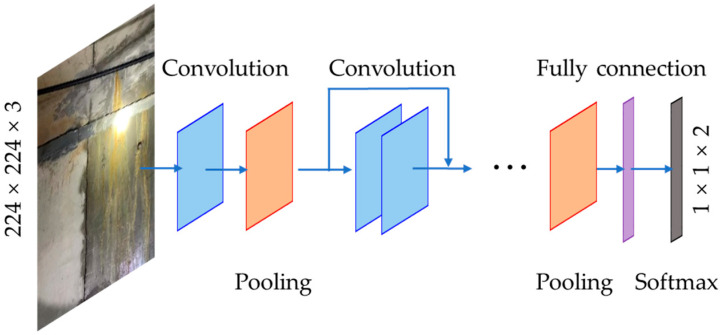
Modified ResNet18 model.

The architecture of the modified ResNet18 model is shown in Figure 4. As shown in this figure, the modified ResNet18 model had many convolutional layers and pooling layers. The last pooling layer was fully connected with the output layer. In the ResNet model, shortcut connections are built between skip layers to deal with the vanishing and exploding gradient problems, so ResNet can be very deep.

### 2.2. Transfer Learning Strategy

**Figure 5:**
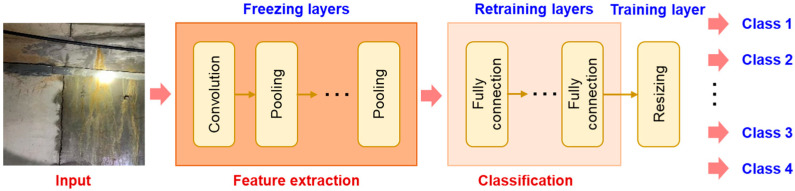
Transfer learning configurations of a deep neural network for water leakage identification.

As mentioned above, all pretrained models were downloaded from the Torchvision library. The models were pretrained on large datasets (e.g., ImageNet). Therefore, these pretrained models had the ability to extract the features from the images. As shown in the examples in Figure 5, in transfer learning, the lower-level layer of the pretrained models was frozen, and the parameters of these layers were not updated in the retraining process. However, to classify the target leakage dataset, the high-level fully connected layers of the VGG16 model and AlexNet model and the resized output layers of the SqueezeNet model and ResNet18 model were retrained using the training data from the target leakage dataset.

In the training of all classification models, the cross-entropy between the prediction distribution and the real distribution was used as the loss function. The cross-entropy was calculated by

(1) $$E\left(x\right)=-\sum_{i=1}^{n}P_{i}\left(x\right)\log Q_{i}^{l}\left(x\right)$$

where $P_{i}\left(x\right)$ is the predicted probability that the sample x belongs to the ith class and $Q_{i}^{l}\left(x\right)$ is the real probability that the sample x belongs to the ith class. The mini-batch gradient descent and RMSProp (root mean square propagation) algorithm were used to update the deep neural networks. The mini-batch size was set to 5, and the learning rate was set to $10^{-5}$. The number of training epochs was determined by k-fold cross-validation.

### 2.3. Dataset Preparation

**Figure 6:**
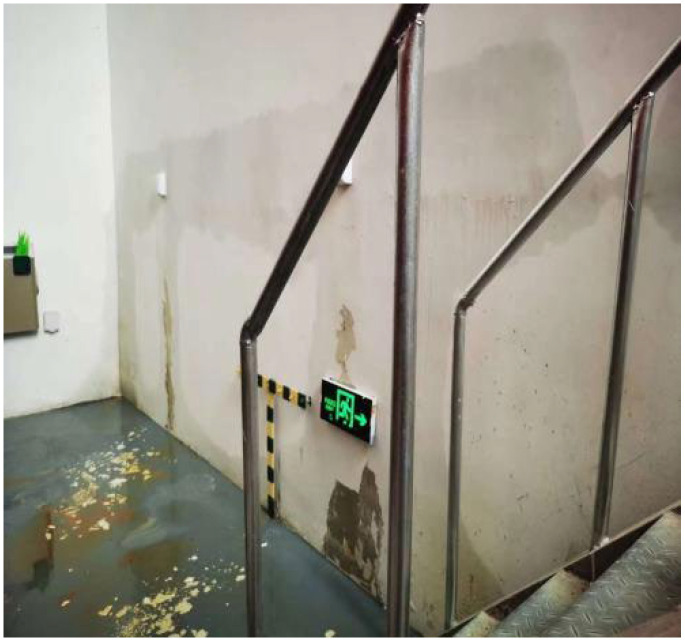
Water leakage image of the underground garage.

**Figure 7:**
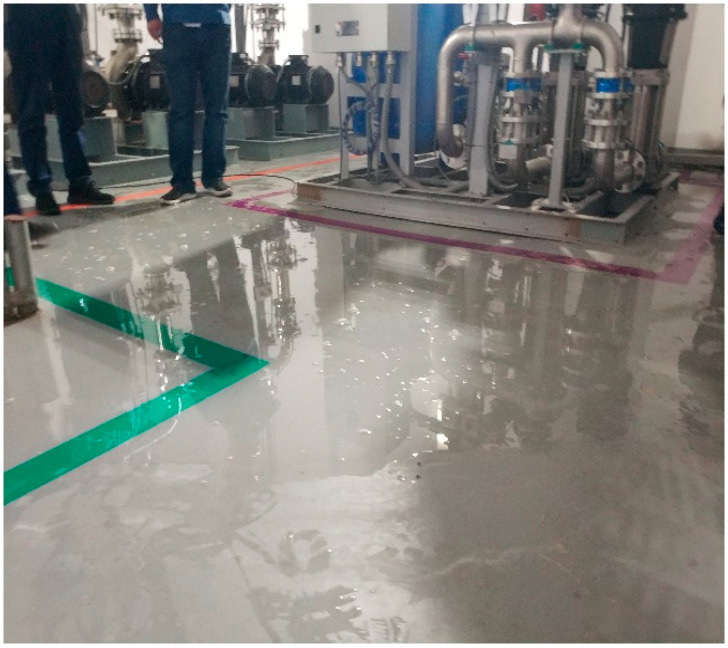
Water leakage image of the underground equipment room.

**Figure 8:**
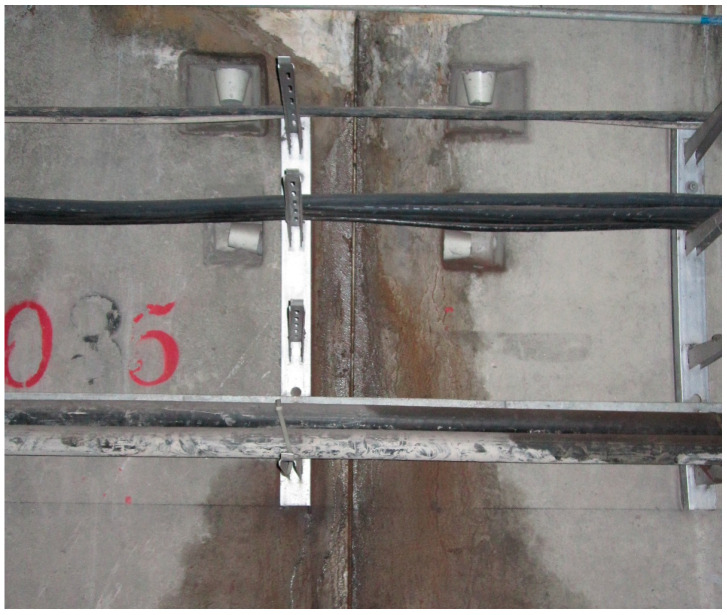
Water leakage image of the underground tunnel lining.

**Figure 9:**
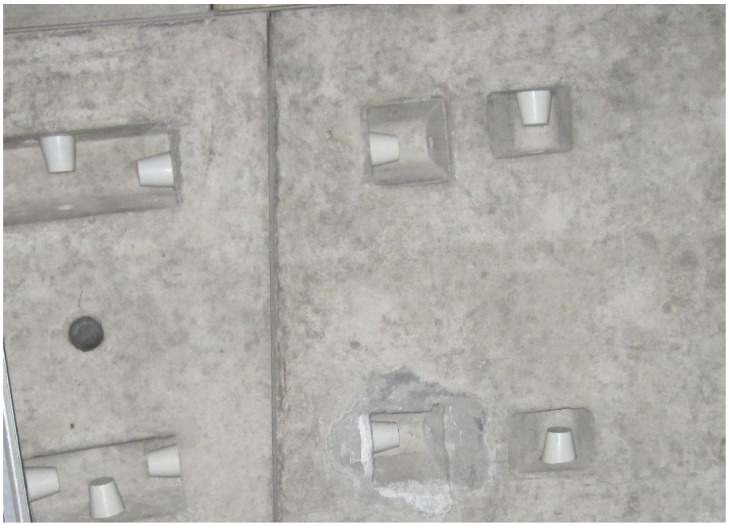
Images of the underground structure without water leakage.

The datasets used in the examples were created through three approaches, including an online search, on-site photography, and an open-source dataset [42]. As shown in Figure 6, Figure 7, Figure 8 and Figure 9, the datasets contained water leakage images of the underground garage, water leakage images of the underground equipment room, water leakage images of the underground tunnel lining, images of underground structures without water leakage, etc. There were 136 high-resolution leakage images collected through an online search and on-site photography. By cropping, flipping, and adding noise, 1431 small-sized leakage images were obtained. The other 4655 leakage images were directly collected from the open-source dataset. By cropping 100 high-resolution no-leakage images of the open-source dataset, 1200 small-sized no-leakage images were obtained. Then, by flipping in two directions and adding noise, the other 6000 small-sized no-leakage images were obtained. Finally, the original datasets were extended to a new dataset with 13,286 images in total. In the new dataset, the number of water leakage images was 6086, and the number of images without water leakage was 7200. All images were in RGB format and had a size of 224 × 224 × 3.

In the experiments, a certain proportion of data was randomly selected as the training set, and the remaining data were used to test the classification performance. To compare the performance of the pretrained models under the different sizes of the training set, the ratio of the training set changed from 0.05 to 0.30 with a step of 0.01, the size of the training set increased from 664 to 3986, and the size of the remaining test set changed from 12,622 to 9300. A detailed configuration of the training set and test set of 26 different experiments is shown in Table 1.

### 2.4. Identification Performance Evaluation Metrics

**Figure 10:**
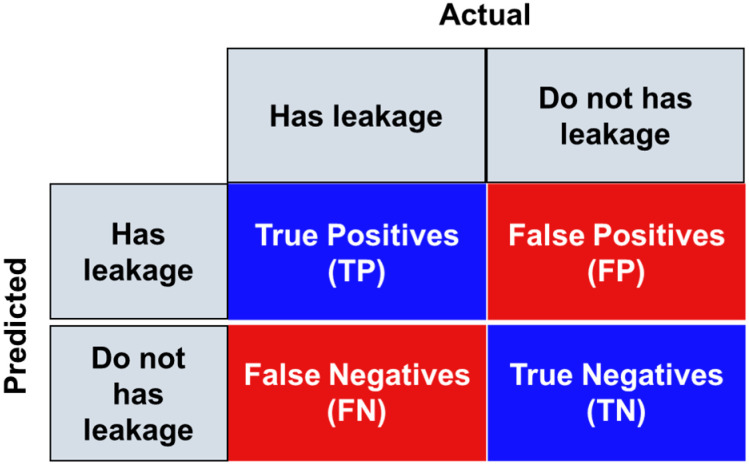
Confusion matrix of leakage classification.

The classification metrics used in this paper included classification accuracy, classification precision, classification recall, and classification F1 score. As shown by the confusion matrix in Figure 10, TP represents the number of leakage images accurately classified as leakage and FP represents the number of no-leakage images mistakenly classified as leakage. Similarly, TN represents the number of no-leakage images accurately classified as no leakage and FN represents the number of leakage images mistakenly classified as no leakage.

The classification metrics were then calculated by

(2) $$Accuracy=\frac{TP+TN}{TP+FP+FN+TN}$$

(3) $$\;Precision=\frac{TP}{TP+FP}$$

(4) $$Recall=\frac{TP}{TP+FN}$$

(5) $$F1-score=2\frac{Precision\cdot Recall}{Precision+Recall}$$

## 3. Experimental Results

To test the impact of transfer learning on the classification performance of deep neural networks, the deep neural networks trained using different sizes of data were used to classify the remaining test set. For each test, both the training methods with and without transfer learning were used. In the method with transfer learning, the deep neural networks and their pretrained parameters were both downloaded from Torchvision library, and the parameters of the low-level layers were not updated in the training process. On the contrary, in the method without transfer learning, the deep neural networks were also downloaded from Torchvision library, but all model parameters were randomly initialized. It is worth noting that the models with and without transfer learning used the same hyperparameter configuration.

### 3.1. Example 1: Test Results of the VGG16 Model

**Figure 11:**
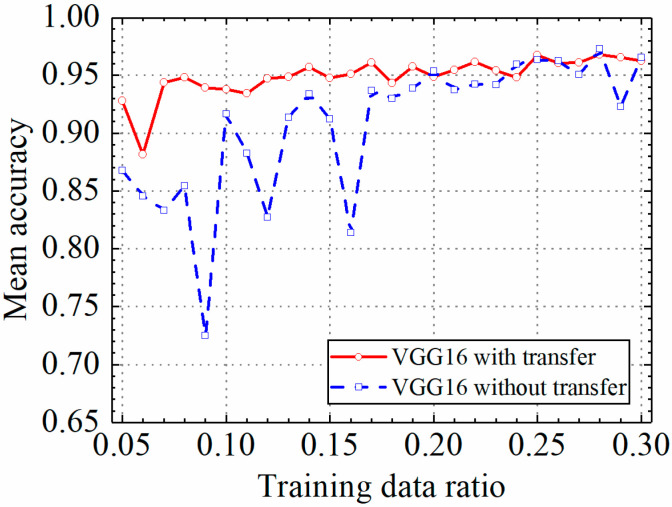
Example 1: prediction accuracy of different methods under different training data ratios.

**Figure 12:**
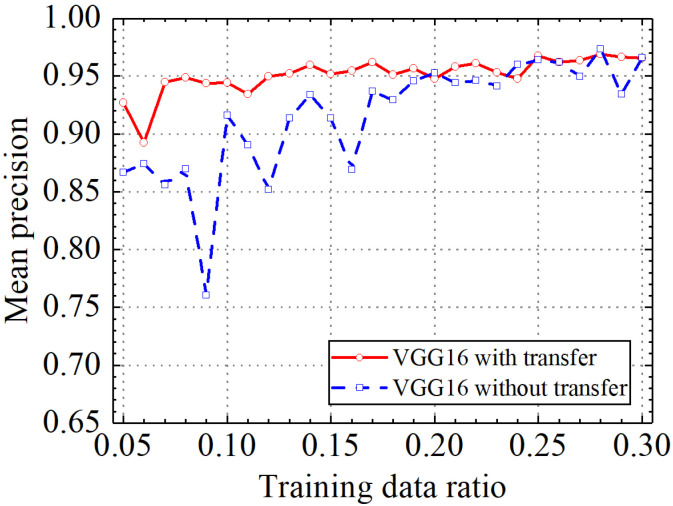
Example 1: prediction precision of different methods under different training data ratios.

**Figure 13:**
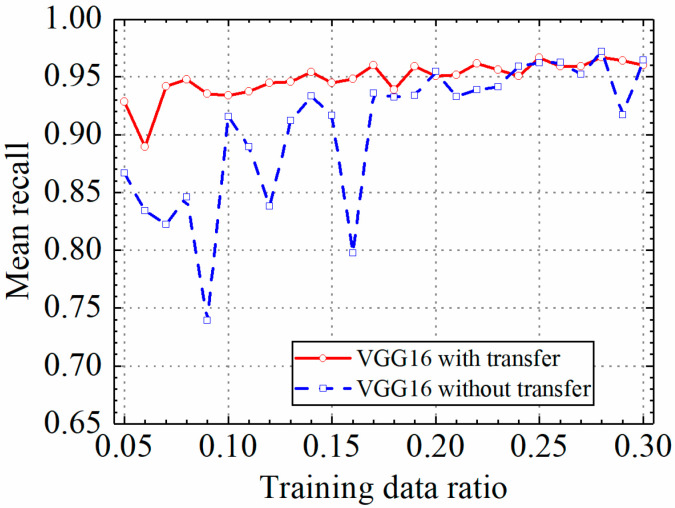
Example 1: prediction recall of different methods under different training data ratios.

**Figure 14:**
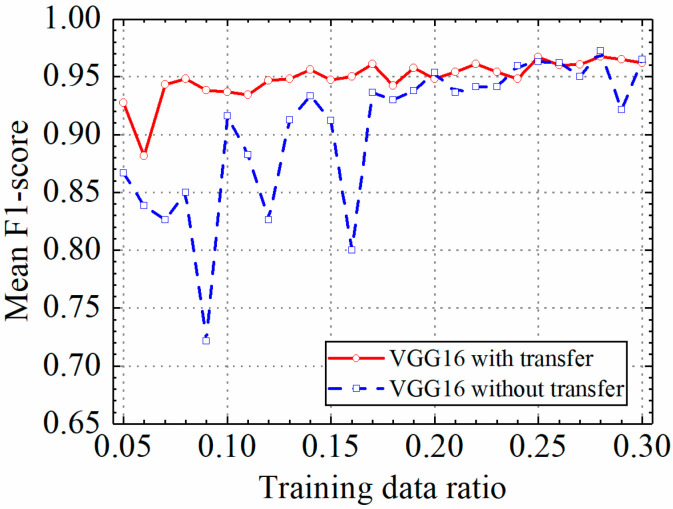
Example 1: prediction F1 score of different methods under different training data ratios.

**Figure 15:**
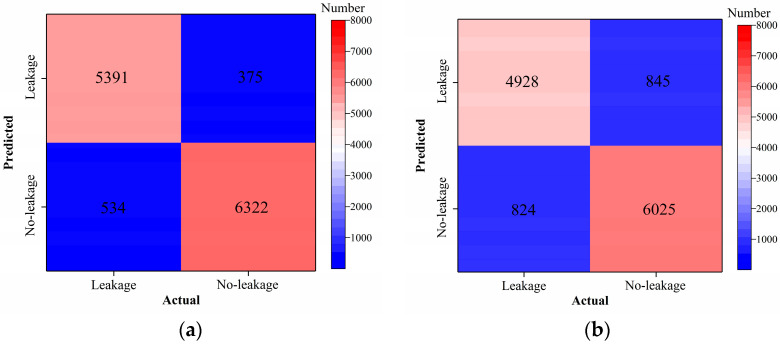
Example 1: heatmap of the confusion matrix of the prediction results when the ratio of training data was 0.05. (**a**) VGG16 with transfer and (**b**) VGG16 without transfer.

In this section, the test results of the VGG16 model are discussed. Table 2 shows the prediction results of the VGG16 model with and without transfer learning. Figure 11, Figure 12, Figure 13 and Figure 14 compare classification accuracy, classification precision, classification recall, and classification F1 score by the VGG16 model with and without transfer learning. As shown in Figure 11, Figure 12, Figure 13 and Figure 14, the classification metrics by the VGG16 model with transfer learning were overall higher than the method without transfer learning. When the ratio of training data was 0.05, classification accuracy, classification precision, classification recall, and classification F1 score by the VGG16 model with transfer learning were 0.928, 0.927, 0.929, and 0.928, respectively, which were higher than 0.868, 0.867, 0.867, and 0.867 by the VGG16 model without transfer learning. The heatmap of the confusion matrix of the prediction results by the VGG16 models when the ratio of training data was 0.05 is shown in Figure 15. With the increase in the ratio of training data, both methods obtained higher classification metrics. However, the increasing process of the VGG16 model with transfer learning was more stable than the VGG16 model without transfer learning.

When the ratio of training data reached 0.2, the classification metrics by the VGG16 model without transfer learning were similar to those of the VGG16 model with transfer learning. When the ratio of training data was 0.3, classification accuracy, classification precision, classification recall, and classification F1 score by the VGG16 model with transfer learning were 0.962, 0.966, 0.960, and 0.962, respectively, and the results by the VGG16 model without transfer learning were 0.965, 0.966, 0.965, and 0.965, respectively. Due to the small randomness of training, the trained VGG16 model without transfer learning achieved slightly better results than the VGG16 model trained with transfer learning. The results showed that transfer learning could significantly improve classification performance of the VGG16 model on leakage defects when the ratio of training data was lower than 0.2 and could deal with the insufficient training data problem.

### 3.2. Example 2: Test Results of the AlexNet Model

**Figure 16:**
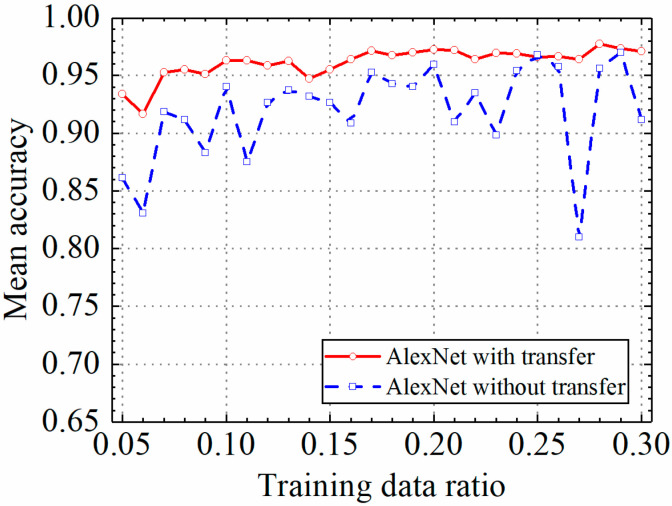
Example 2: prediction accuracy of different methods under different training data ratios.

**Figure 17:**
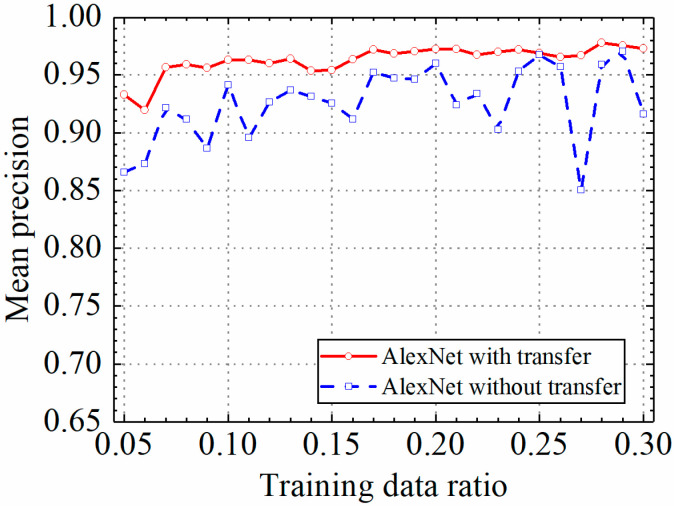
Example 2: prediction precision of different methods under different training data ratios.

**Figure 18:**
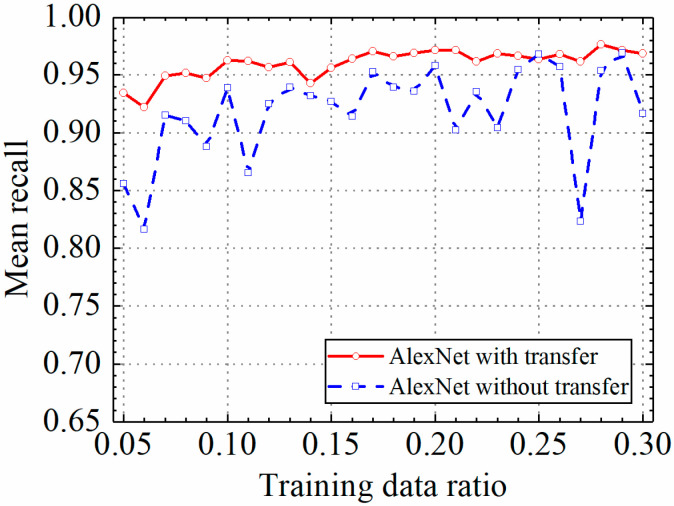
Example 2: prediction recall of different methods under different training data ratios.

**Figure 19:**
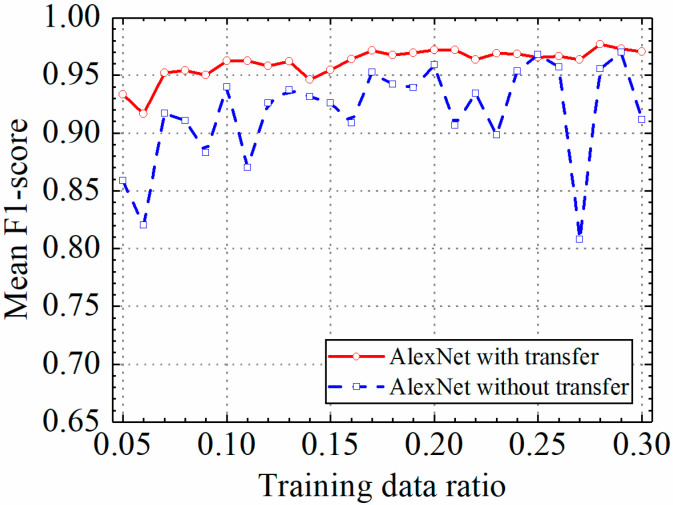
Example 2: prediction F1 score of different methods under different training data ratios.

The test results of the AlexNet model are discussed in this section. The detailed prediction results of the AlexNet model with and without transfer learning are shown in Table 3, and classification accuracy, classification precision, classification recall, and classification F1 score by the AlexNet model with and without transfer learning are shown in Figure 16, Figure 17, Figure 18 and Figure 19.

**Figure 20:**
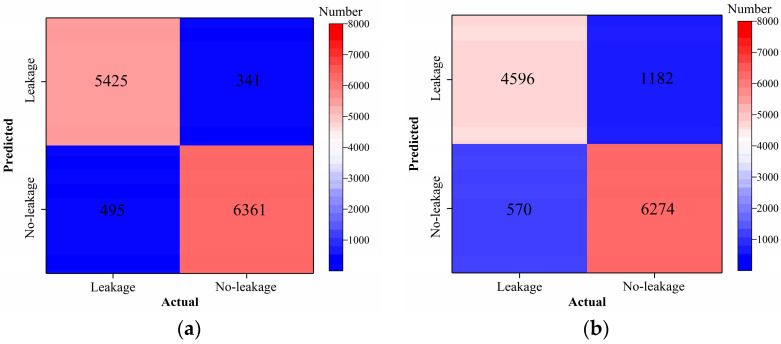
Example 2: heatmap of the confusion matrix of the prediction results when the ratio of training data was 0.05. (**a**) AlexNet with transfer and (**b**) AlexNet without transfer.

As shown in Figure 16, Figure 17, Figure 18 and Figure 19, the classification metrics on the leakage dataset by the AlexNet model with transfer learning were overall higher than the method without transfer learning. When the ratio of training data was 0.05, classification accuracy, classification precision, classification recall, and classification F1 score by the AlexNet model with transfer learning were 0.934, 0.933, 0.934, and 0.933, respectively, which were higher than 0.861, 0.866, 0.856, and 0.859 by the AlexNet model without transfer learning. The heatmap of the confusion matrix of the prediction results by the AlexNet models when the ratio of training data was 0.05 is shown in Figure 20. However, the AlexNet model with transfer learning reached very high classification metrics when the ratio of training data was 0.05. With the increase in the ratio of training data, the classification metrics by the AlexNet model with transfer learning still slightly increased. However, the classification metrics by the AlexNet model without transfer learning were not stable. It changed within a range of approximately 0.85 to 0.95 when the ratio of training data increased.

When the ratio of training data was 0.3, classification accuracy, classification precision, classification recall, and classification F1 score by the AlexNet model with transfer learning were 0.971, 0.973, 0.969, and 0.970, respectively, but the results by the VGG16 model without transfer learning were 0.912, 0.916, 0.917, and 0.912, respectively. The results showed that the AlexNet model with transfer learning could obtain higher classification performance on leakage defects than that without transfer learning. Because of the constant pretrained model parameters of the AlexNet model, the AlexNet model with transfer learning also obtained a more stable classification performance on leakage defects.

### 3.3. Example 3: Test Results of the SqueezeNet Model

**Figure 21:**
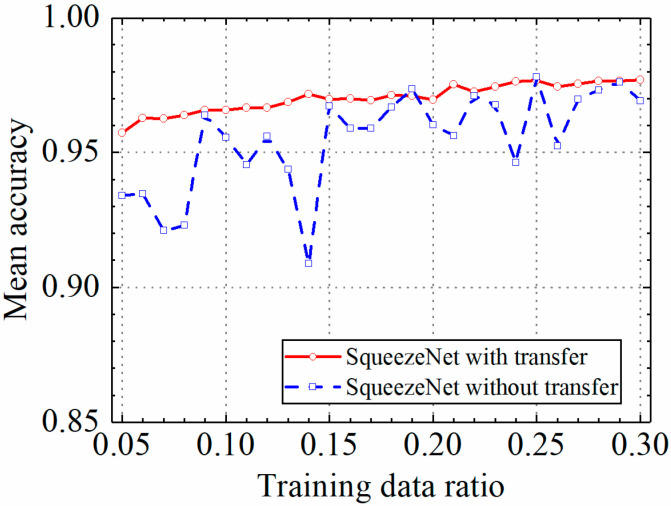
Example 3: prediction accuracy of different methods under different training data ratios.

**Figure 22:**
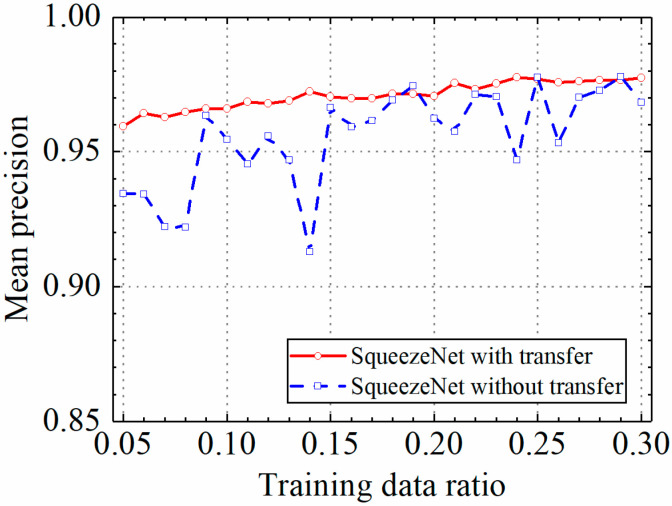
Example 3: prediction precision of different methods under different training data ratios.

**Figure 23:**
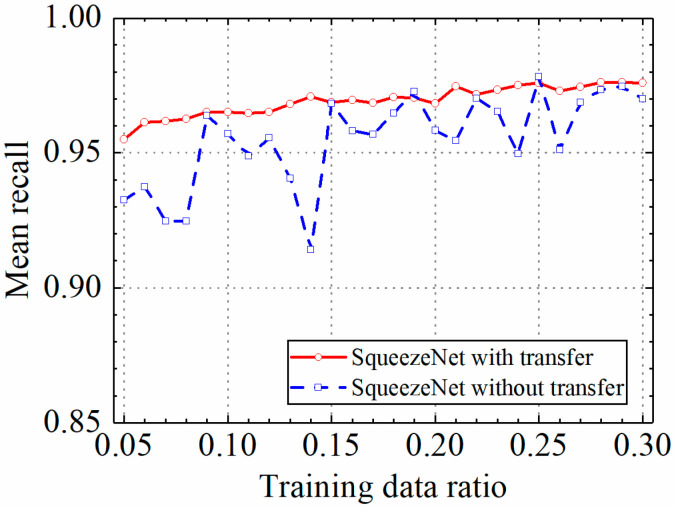
Example 3: prediction recall of different methods under different training data ratios.

**Figure 24:**
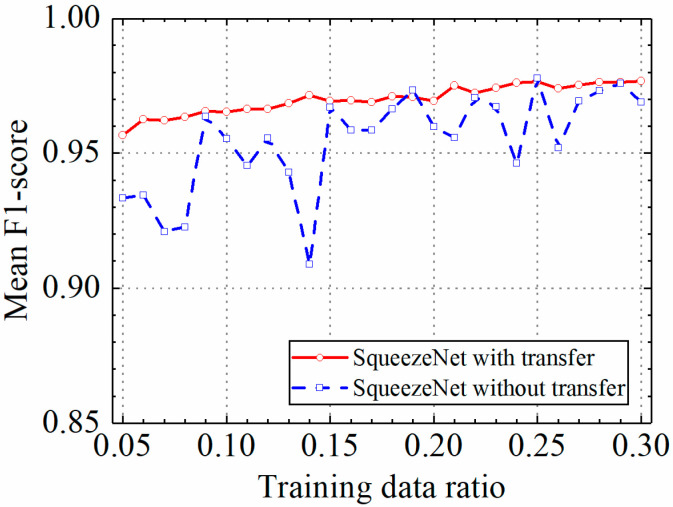
Example 3: prediction F1 score of different methods under different training data ratios.

**Figure 25:**
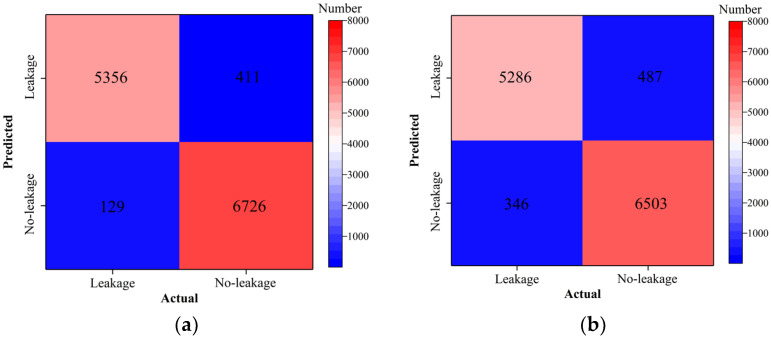
Example 3: heatmap of the confusion matrix of the prediction results when the ratio of training data was 0.05. (**a**) SqueezeNet with transfer and (**b**) SqueezeNet without transfer.

In this section, the test results of the SqueezeNet model are discussed. Table 4 shows the prediction results of the SqueezeNet model with and without transfer learning. Figure 21, Figure 22, Figure 23 and Figure 24 compare classification accuracy, classification precision, classification recall, and classification F1 score by the SqueezeNet model with and without transfer learning. As shown in Figure 21, Figure 22, Figure 23 and Figure 24, the classification metrics by the SqueezeNet model with transfer learning were overall higher than the method without transfer learning. When the ratio of training data was 0.05, classification accuracy, classification precision, classification recall, and classification F1 score by the SqueezeNet model with transfer learning were 0.957, 0.959, 0.955, and 0.957, respectively, which were higher than 0.934, 0.934, 0.933, and 0.934 by the SqueezeNet model without transfer learning. The heatmap of the confusion matrix of the prediction results by the SqueezeNet models when the ratio of training data was 0.05 is shown in Figure 25.

With the increase in the ratio of training data, the classification metrics by the SqueezeNet model with transfer learning increased approximately monotonically. However, the increasing process of the SqueezeNet model without transfer learning was unstable. When the ratio of training data was 0.3, the SqueezeNet models with and without transfer learning obtained similar classification metrics on the leakage dataset, and classification accuracy, classification precision, classification recall, and classification F1 score by the SqueezeNet model with transfer learning were 0.977, 0.977, 0.976, and 0.977, respectively and the results by the SqueezeNet model without transfer learning were 0.969, 0.968, 0.970, and 0.969, respectively. The results showed that using transfer learning, the SqueezeNet model could provide higher and more stable classification metrics on leakage defects.

### 3.4. Example 4: Test Results of the ResNet18 Model

This section presents the test results of the ResNet18 model. The detailed prediction results of the ResNet18 model with and without transfer learning are shown in Table 5.

**Figure 26:**
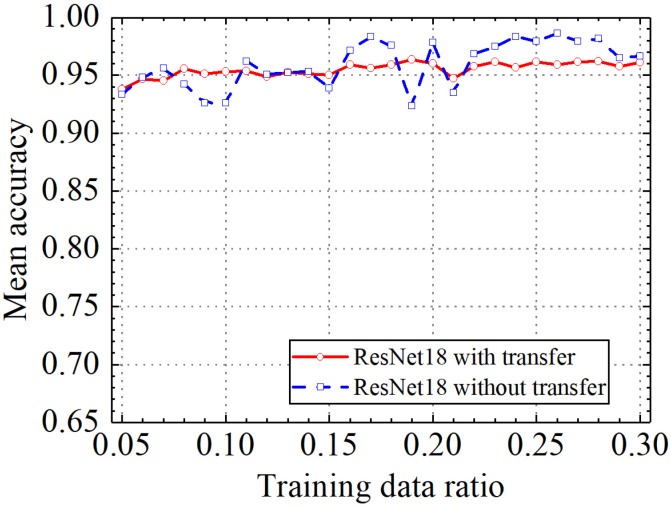
Example 4: prediction accuracy of different methods under different training data ratios.

**Figure 27:**
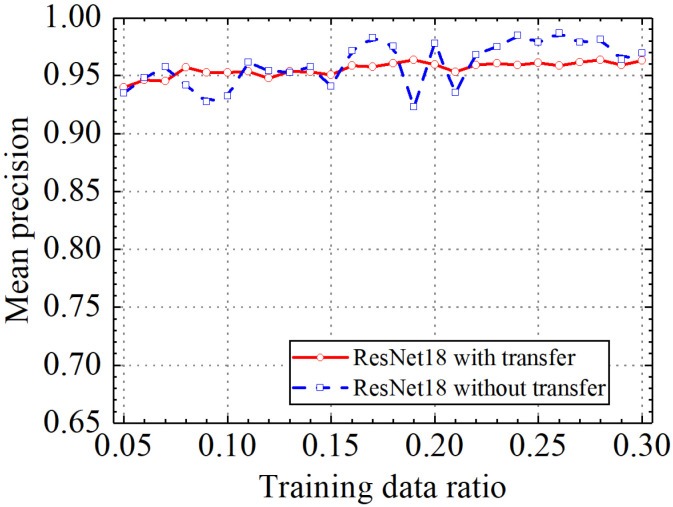
Example 4: prediction precision of different methods under different training data ratios.

**Figure 28:**
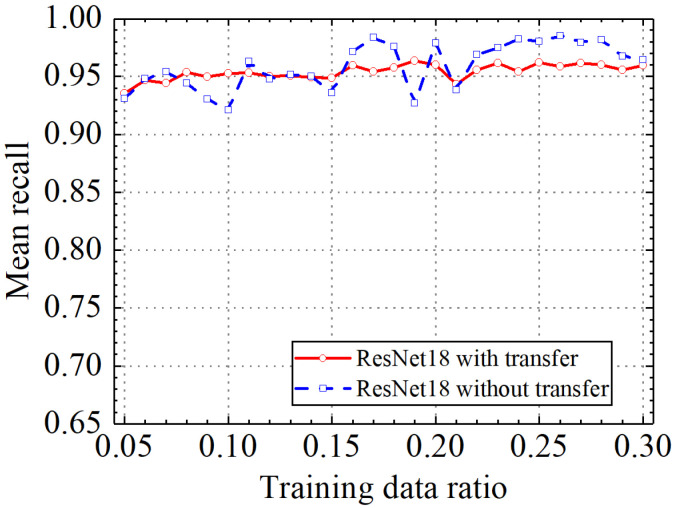
Example 4: prediction recall of different methods under different training data ratios.

**Figure 29:**
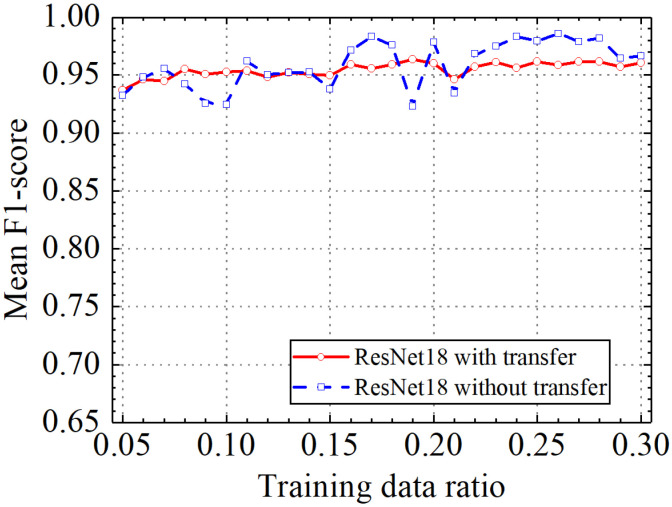
Example 4: prediction F1 score of different methods under different training data ratios.

**Figure 30:**
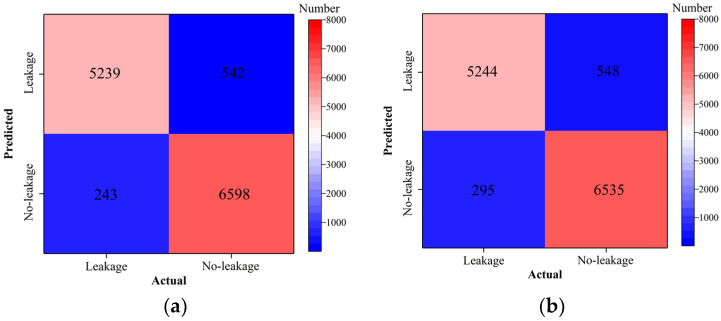
Example 4: heatmap of the confusion matrix of the prediction results when the ratio of training data was 0.05. (**a**) ResNet18 with transfer and (**b**) ResNet18 without transfer.

Classification accuracy, classification precision, classification recall, and classification F1 score by the ResNet18 model with and without transfer learning are shown in Figure 26, Figure 27, Figure 28 and Figure 29. As shown in Figure 26, Figure 27, Figure 28 and Figure 29, the classification metrics on the leakage dataset by the ResNet18 model with transfer learning were overall similar to those without transfer learning. When the ratio of training data was 0.05, classification accuracy, classification precision, classification recall, and classification F1 score by the ResNet18 model with transfer learning were 0.938, 0.940, 0.935, and 0.937, respectively. The prediction results by the ResNet18 model without transfer learning were 0.933, 0.935, 0.931, and 0.933, respectively. The heatmap of the confusion matrix of the prediction results by the ResNet18 model when the ratio of training data was 0.05 is shown in Figure 30. With the increase in the ratio of training data, the classification metrics by the ResNet18 model with transfer learning increased slightly. However, the classification metrics by the ResNet18 model without transfer learning were not stable. When the ratio of training data was 0.3, classification accuracy, classification precision, classification recall, and classification F1 score by the ResNet18 model with transfer learning were 0.961, 0.963, 0.959, and 0.961, respectively, which is similar to the 0.967, 0.969, 0.965, and 0.966 of the ResNet18 model without transfer learning. The results showed that the classification metrics on leakage defects by the ResNet18 model with transfer learning were overall similar to those without transfer learning. This may be because ResNet18 has a specific network structure (e.g., shortcut connection between skip layers), and ResNet18 has strong feature extraction capabilities and can achieve similar performance to ResNet18 with transfer learning using a small amount of training data. However, due to the pretrained model parameters, the prediction results by the ResNet18 model with transfer learning were more stable than the ResNet18 model without transfer learning.

### 3.5. Comparison of the Different Classification Models with Transfer Learning

**Figure 31:**
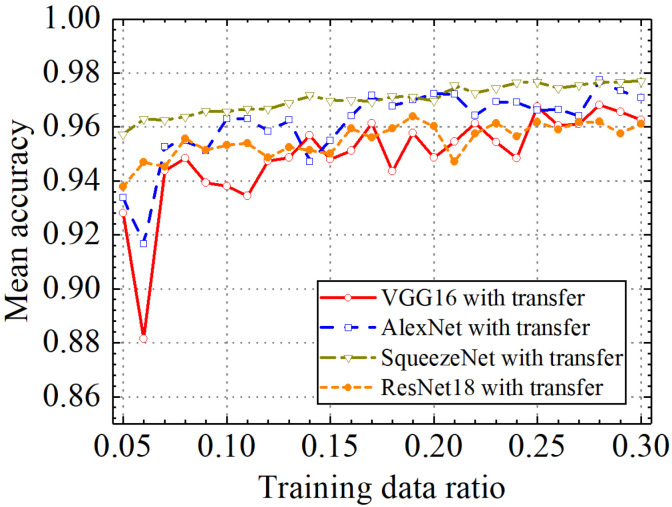
Comparison of mean prediction accuracy by different pretrained models.

**Figure 32:**
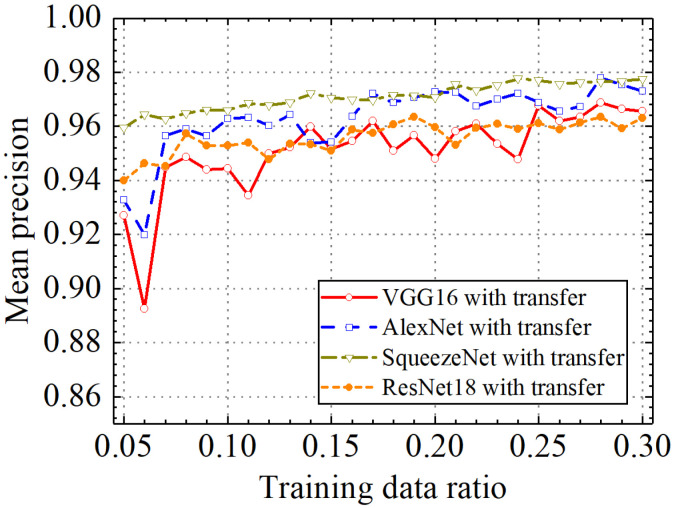
Comparison of mean prediction precision by different pretrained models.

**Figure 33:**
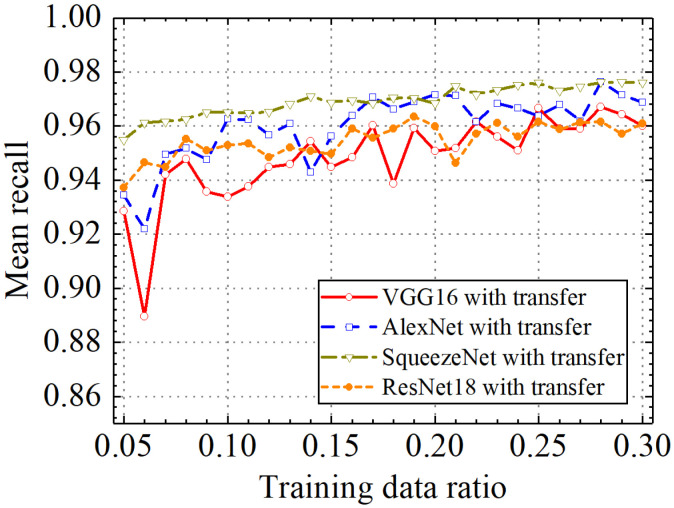
Comparison of mean prediction recall by different pretrained models.

**Figure 34:**
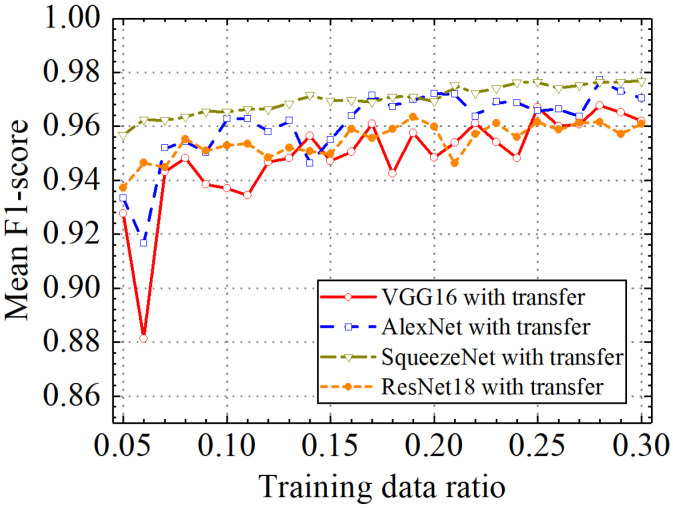
Comparison of mean prediction F1 score by different pretrained models.

In the last section, the classification performance of the different classification models with transfer learning was compared. Figure 31, Figure 32, Figure 33 and Figure 34 show classification accuracy, classification precision, classification recall, and classification F1 score on the leakage dataset by different classification models. As shown in these figures, overall, the SqueezeNet model obtained higher performance than the comparative models on the leakage dataset for all classification metrics.

When the train data ratio was 0.05, the prediction accuracy of the SqueezeNet model was 0.957, which was higher than 0.928, 0.934, and 0.938 of the VGG16, AlexNet, and ResNet18 models. For classification precision, the SqueezeNet model obtained a value of 0.959 when the train data ratio was 0.05, but the values for the VGG16, AlexNet, and ResNet18 models were 0.927, 0.933, and 0.940, respectively. When the train data ratio was 0.05, the obtained classification recall by the SqueezeNet model was 0.955, which was also higher than 0.929, 0.934, and 0.935 of the VGG16, AlexNet, and ResNet18 models. Considering classification F1 score, the prediction value of the SqueezeNet model was 0.957, but the results of the VGG16, AlexNet, and ResNet18 models were 0.928, 0.933, and 0.937, respectively. With the increase in the train data ratio, all models obtained higher classification performance. However, the increased process of classification performance of the SqueezeNet model was more stable than the other models. When the ratio of training data was 0.3, classification accuracy, classification precision, classification recall, and classification F1 score by the VGG16 model with transfer learning were, respectively, 0.962, 0.966, 0.960, and 0.962. Those of the AlexNet model with transfer learning were, respectively, 0.971, 0.973, 0.969, and 0.970. The four classification metrics of the ResNet18 model with transfer learning were, respectively, 0.961, 0.963, 0.959, and 0.961. However, when the ratio of training data was 0.3, classification accuracy, classification precision, classification recall, and classification F1 score by the SqueezeNet model with transfer learning were 0.977, 0.977, 0.976, and 0.977, respectively, which were higher than those of the other methods. The results showed that the SqueezeNet model with transfer learning has a higher classification performance on the leakage defects than the other comparative methods.

**Figure 35:**
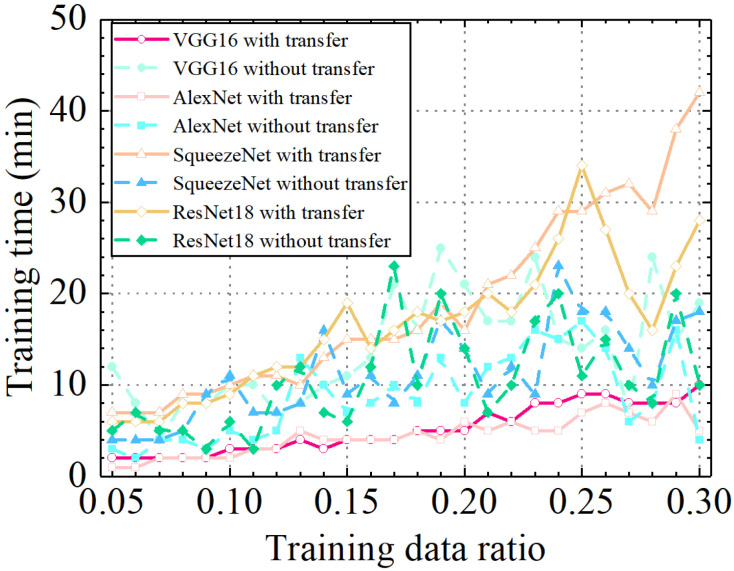
Comparison of the training time of different models under different training data ratios.

To test the computational efficiency of the models, the training time of different final models with and without transfer learning was compared, as shown in Table 6 and Figure 35. There are many factors that affect training time, such as computer equipment, the number of model parameters, training data size, the number of training epochs, etc. The computer equipment used in this paper was a personal computer with an AMD Ryzen 9 5950X 16-Core Processor CPU and an NVIDIA GeForce RTX 3090 GPU. For a specific ratio of training data, the training data sizes for different models were equal, and the number of model parameters and training epochs might be the main factors affecting the training time.

As shown in Table 6 and Figure 35, the average training time for the VGG16 model, AlexNet model, SqueezeNet model, and ResNet model with transfer learning were 5.2 min, 4.4 min, 18.8 min, and 16.5 min, respectively. The average training time for the VGG16 model, AlexNet model, SqueezeNet model, and ResNet model without transfer learning were 13.8 min, 8.8 min, 11.3 min, and 10.6 min, respectively. The results showed that AlexNet achieved the highest computational efficiency, regardless of whether transfer learning was used or not. The VGG16 and AlexNet models with transfer learning used less training time than those without transfer learning. That was because the VGG16 and AlexNet models with transfer learning required a smaller number of training epochs to provide high prediction accuracy. However, the SqueezeNet and ResNet18 models with transfer learning used more training time than those without transfer learning, which may be because the training process of the models without transfer learning was more unstable than the models with transfer learning, and the optimal number of train epochs determined by cross-validation was smaller. As shown in Figure 35, with the increase in the train data ratio, the training time of the models with transfer learning increased. However, the training time of the models without transfer learning was unstable when the train data ratio increased. This phenomenon may also be caused by the unstable training process of the models without transfer learning.

## 4. Conclusions and Discussion

In this paper, a transfer learning strategy was developed to deal with the problems of data shortage in training deep learning-based classification models for underground structure leakage identification. The classification performance of four famous classification models, including VGG16, AlexNet, SqueezeNet, and ResNet18, with the transfer learning on the leakage dataset was comparatively studied under different sizes of training data.

The results showed that the VGG16, AlexNet, and SqueezeNet models with transfer learning could overall provide higher and more stable classification performance on the leakage dataset than those without transfer learning. The ResNet18 model with transfer learning could overall provide a similar value of classification performance on the leakage dataset than that without transfer learning, but its classification performance was more stable than that without transfer learning. When the ratio of training data was 0.05, classification accuracy, classification precision, classification recall, and classification F1 score by the VGG16 model with transfer learning were 0.928, 0.927, 0.929, and 0.928, respectively, and those of the VGG16 model without transfer learning were 0.868, 0.867, 0.867, and 0.867 respectively. The classification metrics by the AlexNet model with transfer learning were 0.934, 0.933, 0.934, and 0.933, respectively, which were higher than 0.861, 0.866, 0.856, and 0.859 by the AlexNet model without transfer learning. The classification metrics by the SqueezeNet model with transfer learning were 0.957, 0.959, 0.955, and 0.957, respectively, and those of the SqueezeNet model without transfer learning were 0.934, 0.934, 0.933, and 0.934. The classification metrics by the ResNet18 model with transfer learning were 0.938, 0.940, 0.935, and 0.937, respectively, and the prediction results by the ResNet18 model without transfer learning were 0.933, 0.935, 0.931, and 0.933, respectively.

In addition, the SqueezeNet model obtained overall higher and more stable performance than the comparative models on the leakage dataset for all classification metrics. When the train data ratio was 0.05, the prediction accuracy of the SqueezeNet model was 0.957, which was higher than 0.928, 0.934, and 0.938 of the VGG16, AlexNet, and ResNet18 models. For the classification precision, the SqueezeNet model obtained a value of 0.959 when the train data ratio was 0.05, but the values for the VGG16, AlexNet, and ResNet18 models were 0.927, 0.933, and 0.940, respectively. The obtained classification recall by the SqueezeNet model was 0.955, which was also higher than 0.929, 0.934, and 0.935 of the VGG16, AlexNet, and ResNet18 models. Considering the classification F1 score, the prediction value of the SqueezeNet model was 0.957, but the results of the VGG16, AlexNet, and ResNet18 models were 0.928, 0.933, and 0.937, respectively.

In the methods with transfer learning, the classification models inherit a strong ability to extract the features of images. During the retraining process in this paper, the feature extraction layers were frozen, and only the weights in the classification layers were updated. The retraining process is actually learning the mapping from extracted features to the classification results. Therefore, the retraining process is generally easier than training the entire classification models without transfer, and less training data are required. In this paper, all the feature extraction layers of the models with transfer learning were fixed during the retraining process. However, if some of the convolution layers are retrained, the classification performance may change. The optimal retraining scheme for different models is worth further research.

## Figures and Tables

**Table 1:** Configuration of the training set and test set of different experiments.

Test Number	Ratio of Training Set	Size of Training Set	Size of Test Set
1	0.05	664	12,622
2	0.06	797	12,489
3	0.07	930	12,356
4	0.08	1063	12,223
5	0.09	1196	12,090
6	0.10	1329	11,957
7	0.11	1461	11,825
8	0.12	1594	11,692
9	0.13	1727	11,559
10	0.14	1860	11,426
11	0.15	1993	11,293
12	0.16	2126	11,160
13	0.17	2259	11,027
14	0.18	2391	10,895
15	0.19	2524	10,762
16	0.20	2657	10,629
17	0.21	2790	10,496
18	0.22	2923	10,363
19	0.23	3056	10,230
20	0.24	3189	10,097
21	0.25	3322	9965
22	0.26	3454	9832
23	0.27	3587	9699
24	0.28	3720	9566
25	0.29	3853	9433
26	0.30	3986	9300

**Table 2:** Example 1: prediction results of the VGG16 model with and without transfer learning.

Ratio	Accuracy		Precision		Recall		F1 Score	
Transfer	Yes	No	Yes	No	Yes	No	Yes	No
0.05	0.928	0.868	0.927	0.867	0.929	0.867	0.928	0.867
0.06	0.881	0.846	0.892	0.874	0.890	0.834	0.881	0.839
0.07	0.944	0.833	0.945	0.856	0.942	0.822	0.943	0.826
0.08	0.948	0.855	0.949	0.870	0.948	0.846	0.948	0.850
0.09	0.939	0.725	0.944	0.761	0.936	0.739	0.938	0.722
0.10	0.938	0.917	0.944	0.916	0.934	0.916	0.937	0.916
0.11	0.934	0.882	0.934	0.890	0.938	0.889	0.934	0.882
0.12	0.947	0.827	0.950	0.852	0.945	0.838	0.947	0.826
0.13	0.949	0.914	0.952	0.914	0.946	0.912	0.948	0.913
0.14	0.957	0.934	0.960	0.934	0.954	0.933	0.956	0.934
0.15	0.948	0.912	0.952	0.914	0.945	0.916	0.947	0.912
0.16	0.951	0.814	0.955	0.869	0.948	0.798	0.950	0.800
0.17	0.961	0.937	0.962	0.937	0.960	0.936	0.961	0.936
0.18	0.944	0.930	0.951	0.929	0.939	0.933	0.942	0.930
0.19	0.958	0.939	0.957	0.946	0.959	0.934	0.958	0.938
0.20	0.949	0.954	0.948	0.953	0.951	0.955	0.948	0.954
0.21	0.955	0.937	0.958	0.944	0.952	0.933	0.954	0.936
0.22	0.961	0.942	0.961	0.946	0.961	0.939	0.961	0.942
0.23	0.954	0.942	0.953	0.941	0.956	0.941	0.954	0.941
0.24	0.948	0.960	0.948	0.960	0.951	0.959	0.948	0.959
0.25	0.967	0.963	0.968	0.964	0.967	0.962	0.967	0.963
0.26	0.960	0.962	0.962	0.962	0.959	0.962	0.960	0.962
0.27	0.961	0.951	0.963	0.950	0.959	0.952	0.961	0.950
0.28	0.968	0.973	0.969	0.973	0.967	0.972	0.968	0.973
0.29	0.965	0.923	0.966	0.934	0.964	0.917	0.965	0.921
0.30	0.962	0.965	0.966	0.966	0.960	0.965	0.962	0.965

**Table 3:** Example 2: prediction results of the AlexNet model with and without transfer learning.

Ratio	Accuracy		Precision		Recall		F1 Score	
Transfer	Yes	No	Yes	No	Yes	No	Yes	No
0.05	0.934	0.861	0.933	0.866	0.934	0.856	0.933	0.859
0.06	0.917	0.831	0.920	0.873	0.922	0.816	0.917	0.820
0.07	0.953	0.918	0.957	0.921	0.949	0.915	0.952	0.917
0.08	0.955	0.912	0.959	0.912	0.952	0.910	0.954	0.911
0.09	0.951	0.883	0.956	0.886	0.947	0.888	0.950	0.883
0.10	0.963	0.940	0.963	0.942	0.963	0.939	0.963	0.940
0.11	0.963	0.875	0.963	0.896	0.962	0.865	0.963	0.870
0.12	0.958	0.926	0.960	0.927	0.957	0.925	0.958	0.926
0.13	0.963	0.937	0.964	0.937	0.961	0.940	0.962	0.937
0.14	0.947	0.932	0.954	0.931	0.943	0.932	0.946	0.932
0.15	0.955	0.927	0.954	0.926	0.956	0.927	0.955	0.926
0.16	0.964	0.909	0.964	0.912	0.964	0.914	0.964	0.909
0.17	0.972	0.953	0.972	0.952	0.971	0.953	0.971	0.953
0.18	0.968	0.943	0.969	0.947	0.966	0.940	0.967	0.942
0.19	0.970	0.940	0.971	0.946	0.969	0.936	0.970	0.939
0.20	0.972	0.959	0.973	0.960	0.971	0.958	0.972	0.959
0.21	0.972	0.909	0.973	0.924	0.971	0.902	0.972	0.907
0.22	0.964	0.935	0.967	0.934	0.961	0.935	0.964	0.935
0.23	0.969	0.898	0.970	0.903	0.968	0.904	0.969	0.898
0.24	0.969	0.954	0.972	0.953	0.967	0.955	0.969	0.954
0.25	0.966	0.968	0.969	0.967	0.964	0.968	0.966	0.968
0.26	0.966	0.958	0.966	0.957	0.968	0.957	0.966	0.957
0.27	0.964	0.810	0.967	0.850	0.962	0.823	0.964	0.808
0.28	0.977	0.956	0.978	0.959	0.976	0.954	0.977	0.956
0.29	0.973	0.970	0.975	0.971	0.971	0.969	0.973	0.970
0.30	0.971	0.912	0.973	0.916	0.969	0.917	0.970	0.912

**Table 4:** Example 3: prediction results of the SqueezeNet model with and without transfer learning.

Ratio	Accuracy		Precision		Recall		F1 Score	
Transfer	Yes	No	Yes	No	Yes	No	Yes	No
0.05	0.957	0.934	0.959	0.934	0.955	0.933	0.957	0.933
0.06	0.963	0.935	0.964	0.934	0.961	0.937	0.962	0.935
0.07	0.963	0.921	0.963	0.922	0.962	0.925	0.962	0.921
0.08	0.964	0.923	0.965	0.922	0.962	0.925	0.963	0.923
0.09	0.966	0.964	0.966	0.963	0.965	0.964	0.965	0.964
0.10	0.966	0.956	0.966	0.955	0.965	0.957	0.965	0.955
0.11	0.967	0.945	0.969	0.945	0.965	0.949	0.966	0.945
0.12	0.967	0.956	0.968	0.956	0.965	0.956	0.966	0.956
0.13	0.969	0.944	0.969	0.947	0.968	0.941	0.969	0.943
0.14	0.972	0.909	0.972	0.913	0.971	0.914	0.971	0.909
0.15	0.970	0.967	0.970	0.966	0.969	0.968	0.969	0.967
0.16	0.970	0.959	0.970	0.959	0.969	0.958	0.970	0.959
0.17	0.969	0.959	0.970	0.961	0.968	0.957	0.969	0.959
0.18	0.971	0.967	0.972	0.969	0.971	0.965	0.971	0.966
0.19	0.971	0.974	0.971	0.974	0.970	0.973	0.971	0.973
0.20	0.970	0.960	0.971	0.962	0.968	0.958	0.969	0.960
0.21	0.975	0.956	0.976	0.957	0.975	0.955	0.975	0.956
0.22	0.973	0.971	0.973	0.971	0.972	0.970	0.972	0.971
0.23	0.974	0.968	0.975	0.970	0.973	0.965	0.974	0.967
0.24	0.976	0.946	0.978	0.947	0.975	0.950	0.976	0.946
0.25	0.977	0.978	0.977	0.978	0.976	0.978	0.976	0.978
0.26	0.974	0.953	0.976	0.953	0.973	0.951	0.974	0.952
0.27	0.975	0.970	0.976	0.970	0.975	0.969	0.975	0.969
0.28	0.976	0.973	0.976	0.973	0.976	0.973	0.976	0.973
0.29	0.977	0.976	0.977	0.978	0.976	0.975	0.976	0.976
0.30	0.977	0.969	0.977	0.968	0.976	0.970	0.977	0.969

**Table 5:** Example 4: prediction results of the ResNet18 model with and without transfer learning.

Ratio	Accuracy		Precision		Recall		F1 Score	
Transfer	Yes	No	Yes	No	Yes	No	Yes	No
0.05	0.938	0.933	0.940	0.935	0.935	0.931	0.937	0.933
0.06	0.947	0.948	0.946	0.948	0.947	0.948	0.946	0.948
0.07	0.945	0.956	0.945	0.957	0.944	0.954	0.945	0.956
0.08	0.956	0.942	0.957	0.942	0.954	0.944	0.955	0.942
0.09	0.951	0.926	0.953	0.927	0.950	0.931	0.951	0.926
0.10	0.953	0.926	0.953	0.932	0.953	0.921	0.953	0.925
0.11	0.954	0.962	0.954	0.961	0.953	0.963	0.954	0.962
0.12	0.949	0.951	0.948	0.954	0.950	0.948	0.948	0.950
0.13	0.953	0.952	0.953	0.952	0.951	0.952	0.952	0.952
0.14	0.951	0.953	0.953	0.958	0.949	0.950	0.951	0.953
0.15	0.950	0.939	0.951	0.941	0.949	0.936	0.950	0.938
0.16	0.959	0.972	0.959	0.971	0.960	0.972	0.959	0.971
0.17	0.956	0.983	0.957	0.983	0.954	0.984	0.956	0.983
0.18	0.959	0.976	0.961	0.976	0.958	0.976	0.959	0.976
0.19	0.964	0.923	0.963	0.923	0.964	0.927	0.963	0.923
0.20	0.960	0.978	0.960	0.978	0.960	0.979	0.960	0.978
0.21	0.947	0.935	0.953	0.935	0.943	0.939	0.946	0.935
0.22	0.958	0.969	0.959	0.968	0.956	0.969	0.957	0.968
0.23	0.961	0.975	0.961	0.975	0.961	0.975	0.961	0.975
0.24	0.957	0.983	0.959	0.985	0.954	0.982	0.956	0.983
0.25	0.962	0.980	0.961	0.979	0.962	0.980	0.962	0.979
0.26	0.959	0.986	0.959	0.987	0.959	0.985	0.959	0.986
0.27	0.962	0.979	0.961	0.979	0.961	0.979	0.961	0.979
0.28	0.962	0.982	0.963	0.981	0.960	0.982	0.961	0.982
0.29	0.958	0.965	0.959	0.964	0.956	0.967	0.957	0.965
0.30	0.961	0.967	0.963	0.969	0.959	0.965	0.961	0.966

**Table 6:** Training time of different models under different training data ratios (min).

Ratio	VGG16		AlexNet		SqueezeNet		ResNet18	
Transfer	Yes	No	Yes	No	Yes	No	Yes	No
0.05	2	12	1	3	7	4	6	5
0.06	2	8	1	2	7	4	6	7
0.07	2	4	2	4	7	4	6	5
0.08	2	8	2	4	9	5	8	5
0.09	2	8	2	3	9	9	8	3
0.10	3	10	2	5	10	11	9	6
0.11	3	10	3	4	11	7	11	3
0.12	3	7	3	5	11	7	12	10
0.13	4	8	5	13	10	8	12	12
0.14	3	10	4	10	13	16	15	7
0.15	4	11	4	7	15	9	19	6
0.16	4	13	4	8	15	11	14	12
0.17	4	21	4	10	15	8	16	23
0.18	5	16	5	8	16	11	18	10
0.19	5	25	4	13	19	17	17	20
0.20	5	21	6	8	16	14	18	14
0.21	7	17	5	12	21	9	20	7
0.22	6	17	6	13	22	12	18	10
0.23	8	24	5	16	25	9	21	17
0.24	8	15	5	15	29	23	26	20
0.25	9	14	7	17	29	18	34	11
0.26	9	16	8	14	31	18	27	15
0.27	8	7	7	6	32	14	20	10
0.28	8	24	6	8	29	10	16	8
0.29	8	15	9	16	38	17	23	20
0.30	10	19	5	4	42	18	28	10
Average	5.2	13.8	4.4	8.8	18.8	11.3	16.5	10.6

## Data Availability

Data supporting the reported results can be provided by the corresponding author upon reasonable request.
